# Associations between pretherapeutic body mass index, outcome, and cytogenetic abnormalities in pediatric acute myeloid leukemia

**DOI:** 10.1002/cam4.2554

**Published:** 2019-09-18

**Authors:** Ditte J. A. Løhmann, Peter H. Asdahl, Jonas Abrahamsson, Shau‐Yin Ha, Ólafur G. Jónsson, Gertjan J. L. Kaspers, Minna Koskenvuo, Birgitte Lausen, Barbara De Moerloose, Josefine Palle, Bernward Zeller, Lillian Sung, Henrik Hasle

**Affiliations:** ^1^ Department of Pediatrics and Adolescent Medicine Aarhus University Hospital Aarhus N Denmark; ^2^ Department of Pediatrics Institution for Clinical Sciences Queen Silvia Children's Hospital Gothenburg Sweden; ^3^ Department of Pediatrics Queen Mary Hospital and Hong Kong Pediatric Hematology and Oncology Study Group (HKPHOSG) Hong Kong China; ^4^ Department of Pediatrics Landspitali University Hospital Reykjavik Iceland; ^5^ Department of Pediatrics VU University Medical Center Amsterdam The Netherlands; ^6^ Princess Máxima Center for Pediatric Oncology Utrecht The Netherlands; ^7^ Dutch Childhood Oncology Group The Hague The Netherlands; ^8^ Division of Hematology‐Oncology and Stem Cell Transplantation Children's Hospital and Helsinki University Central Hospital Helsinki Finland; ^9^ Department of Pediatrics and Adolescent Medicine Rigshospitalet University of Copenhagen Copenhagen Denmark; ^10^ Department of Pediatrics Ghent University Hospital Ghent Belgium; ^11^ Department of Woman's and Children's Health Uppsala University Uppsala Sweden; ^12^ Division of Pediatric and Adolescent Medicine Oslo University Hospital Oslo Norway; ^13^ Division of Hematology/Oncology The Hospital for Sick Children Toronto Canada

**Keywords:** acute myeloid leukemia, body mass index, cytogenetic aberrations, mortality, pediatrics, recurrence

## Abstract

**Background:**

Associations between body mass index (BMI), outcome, and leukemia‐related factors in children with acute myeloid leukemia (AML) remain unclear. We investigated associations between pretherapeutic BMI, cytogenetic abnormalities, and outcome in a large multinational cohort of children with AML.

**Methods:**

We included patients, age 2‐17 years, diagnosed with de novo AML from the five Nordic countries (2004‐2016), Hong Kong (2007‐2016), the Netherlands and Belgium (2010‐2016), and Canada and USA (1995‐2012). BMI standard deviations score for age and sex was calculated and categorized according to the World Health Organization. Cumulative incidence functions, Kaplan‐Meier estimator, Cox regression, and logistic regression were used to investigate associations.

**Results:**

In total, 867 patients were included. The median age was 10 years (range 2‐17 years). At diagnosis, 32 (4%) were underweight, 632 (73%) were healthy weight, 127 (15%) were overweight, and 76 (9%) were obese. There was no difference in relapse risk, treatment‐related mortality or overall mortality across BMI groups. The frequency of t(8;21) and inv(16) increased with increasing BMI. For obese patients, the sex, age, and country adjusted odds ratio of having t(8;21) or inv(16) were 1.9 (95% confidence interval (CI) 1.1‐3.4) and 2.8 (95% CI 1.3‐5.8), respectively, compared to healthy weight patients.

**Conclusions:**

This study did not confirm previous reports of associations between overweight and increased treatment‐related or overall mortality in children. Obesity was associated with a higher frequency of t(8;21) and inv(16). AML cytogenetics appear to differ by BMI status.

## Graphical abstract

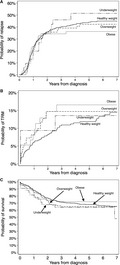

## 1 INTRODUCTION

Acute myeloid leukemia (AML) in children is a clinically and genetically heterogeneous disease with a long‐term overall survival of around 70%, and few known causative factors.^1^, ^2^ Overweight in children is increasing globally and, as a consequence, so are overweight‐related diseases.^3^ Among children with AML, it is important to understand if overweight patients have different outcomes and leukemia‐related features. Overweight at diagnosis has been associated with inferior outcome in pediatric AML in two North American studies,^4^, ^5^ but in a recent study on mainly Nordic patients, we failed to confirm this association.^6^ Likewise, results from studies on the effect of pretherapeutic body mass‐index (BMI) in adults with AML have been conflicting.^7^, ^8^, ^9^ In our previous study, we saw indications of cytogenetic abnormalities being associated with weight‐group,^6^ an association that, to our knowledge, has not been reported in children before.

In this study, we aimed to investigate associations between pretherapeutic BMI and outcome in a large, multinational cohort of children diagnosed with AML and treated on several protocols. Second, we aimed to confirm a possible association between BMI group and cytogenetic abnormalities as suggested by our previous study.

## 2 MATERIALS AND METHODS

### 2.1 Patients

**Figure 1:**
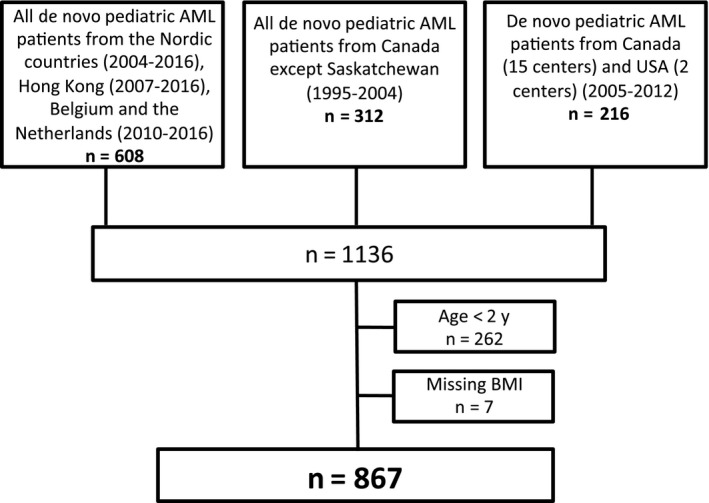
Study cohort and exclusions. The figure shows the included and excluded patients. The cohort was population based in all the specified time periods except for patients from Canada and USA diagnosed from 2005 to 2012. Patients with Down syndrome, acute promyelocytic leukemia or isolated granulocytic sarcoma were not included

We included patients less than 18 years of age diagnosed with de novo AML before November 1st 2016, but without Down syndrome, acute promyelocytic leukemia, or isolated granulocytic sarcoma. Patients originated from the five Nordic countries (Sweden, Denmark, Norway, Finland and Iceland), the Netherlands, Belgium, Hong Kong (NOPHO‐DBH cohort) and Canada and USA. Included patients from the Nordic countries were diagnosed from 2004 to 2016, Hong Kong from 2007 to 2016, Belgium and the Netherlands from 2010 to 2016, Canada from 1995 to 2012 and USA from 2005 to 2012. Included patients from Canada and USA diagnosed from 2005 to 2012 came from a prospective study on host genome variants and infection.^10^ We excluded patients below 2 years of age (BMI calculations in this age group is not standardized), and patients with missing data on BMI at diagnosis (Figure 1).

### 2.2 Data collection and registration

In the NOPHO‐DBH cohort data on demographics, cytogenetic abnormalities, and follow‐up information were prospectively registered by the treating center. In Canada and USA, clinical research associates conducted site visits in order to abstract the required data from medical records. Centers were contacted at later time points to update follow‐up information. The Research Ethics Boards at all participating institutions/countries approved data collection and written informed consent was obtained from all patients/parents/guardians. For Canadian children diagnosed between 1995 and 2004, the need for informed consent was waived given the retrospective nature of the study.

### 2.3 Definitions and statistics

BMI was calculated using height and weight measured at time of diagnosis. BMI standard deviation (SD) score for age and sex was calculated and categorized according to the World Health Organization Child Growth Standards^11^ as following: underweight: <−2 SD, healthy weight: −2 up to +2 SD for age 2‐5 and −2 up to +1 SD for age 6‐17, overweight: >2 up to 3 SD for age 2‐5 and >1 up to 2 SD for age 6‐17, and obese: >3 SD for age 2‐5 and >2 SD for age 6‐17. For all analyses, the healthy weight group was used as reference.

Treatment‐related mortality was defined as death not related to disease progression.^12^ Patients with unknown reason for death were excluded from treatment‐related mortality analyses. Associations between BMI groups and relapse, treatment‐related mortality, and overall survival were investigated using the cumulative incidence function with pseudo‐observations under competing risks and the Kaplan‐Meier estimator. Death was included as a competing risk in the calculation of cumulative incidence of relapse, and death from progressive disease in the calculation of cumulative incidence of treatment‐related mortality. Multiple Cox regression was used to compare groups. Follow‐up started at diagnosis and ended at event, death, or last follow‐up. Cases were censored on November 5, 2016, 6 months before data cutoff (May 5, 2017) to ensure independent censoring (relapse and death could be reported sooner than ongoing follow‐up for patients in continuing remission).

Inv(16)/t(16;16) (in the following referred to as inv(16)), t(8;21) and *KMT2A* rearrangements were selected for the study due to these abnormalities being common and with prognostic significance.^1^, ^13^ Core‐binding factor AML, t(8;21) and inv(16), has generally been associated with a favorable prognosis, whereas the prognostic significance of *KMT2A* rearrangements depends on the fusion partner.^1^ Logistic regression was used to investigate associations between BMI groups and cytogenetic abnormalities. Cases with missing data on cytogenetic abnormalities were excluded from analyses.

BMI standard deviation score as a continuous variable and outcomes were modeled using restricted cubic splines with four knots at BMI SD score −2, 0, 1, and 2.^14^

Confounders incorporated into statistical models were defined as factors, which could influence both the independent variable (BMI group) and dependent variables (treatment‐related mortality, relapse, survival, and cytogenetic abnormalities). Age, sex, and country group (Nordic countries, Belgium/the Netherlands, Hong Kong or Canada/USA) was included in all adjusted models. Year of diagnosis was included in the adjusted models for treatment‐related mortality, relapse, and overall mortality.

## 3 RESULTS

A total of 867 patients included were treated in 10 different countries (Canada n = 394, Sweden n = 116, the Netherlands n = 92, Denmark n = 53, Finland n = 51, Hong Kong n = 50, Belgium n = 48, Norway n = 41, USA n = 17, and Iceland n = 5). Of included cases, n = 692 (80%) were population based, and n = 239 (28%) were included in our previously study of associations between BMI group and outcome.^6^ The median age of diagnosis was 10 years of age (range: 2‐17 years), 53% of cases were male and the median follow‐up time for cases alive at end of follow‐up (n = 615) was 3.9 years (range: 0.1‐13.3 years). Patients were treated on 17 different protocols with AAML0531,^15^ COG9421,^16^ NOPHO‐AML 2004,^17^ DB AML‐01,^18^ and NOPHO‐DBH AML 2012 (http://ClinicalTrials.gov identifier: NCT01828489, ongoing accrual) accounting for 79%. Table 1 shows baseline characteristics according to BMI group. The frequency of underweight and obesity was higher among males, and the median age was higher for the nonhealthy BMI groups. The frequency of obesity was higher in the North American countries.

**Table 1:** Baseline characteristics according to body mass index group

	Total cohort	Underweight N (%)	Healthy weight N (%)	Overweight N (%)	Obese N (%)
Patients	867 (100)	32 (4)	632 (73)	127 (15)	76 (9)
Sex					
Male	462 (53)	22 (5)	319 (69)	68 (15)	53 (11)
Female	405 (47)	10 (2)	313 (77)	59 (15)	23 (6)
Age					
Median (range)	10 (2‐17)	12 (2‐17)	9 (2‐17)	12 (2‐17)	13 (2‐17)
Country group					
The Nordic countries	266 (31)	11 (4)	210 (79)	28 (11)	17 (6)
The Netherlands and Belgium	140 (16)	6 (4)	111 (79)	17 (12)	6 (4)
Hong Kong	50 (6)	5 (10)	35 (70)	7 (14)	3 (6)
Canada and USA	411 (47)	10 (2)	276 (67)	75 (18)	50 (12)
WBC at diagnosis^a^					
0‐9.9	300 (35)	9 (3)	223 (74)	44 (15)	24 (8)
10‐99.9	428 (49)	14 (3)	308 (72)	62 (14)	44 (10)
≥100	137 (16)	9 (7)	99 (72)	21 (15)	8 (6)
Year of diagnosis					
1995‐1999	117 (13)	4 (3)	80 (68)	18 (15)	15 (13)
2000‐2004	130 (15)	2 (3)	85 (65)	26 (20)	16 (12)
2005‐2009	240 (28)	9 (4)	172 (72)	35 (15)	24 (10)
2010‐2014	303 (35)	13 (4)	231 (76)	42 (14)	17 (6)
2015‐2016	77 (9)	3 (4)	64 (83)	6 (8)	4 (5)
Stem cell transplanted in first complete remission	224 (26)	8 (25)	166 (26)	32 (25)	18 (24)

Note

Underweight: <−2 SD, Healthy weight: −2 to +2 SD for age 2‐5 and −2 to +1 SD for age 6‐17, Overweight: >2‐3 SD for age 2‐5 and >1‐2 SD for age 6‐17, Obesity: >3 SD for age 2‐5 and >2 SD for age 6‐17.

Abbreviation: WBC, white blood count.

a 2 missing.

### 3.1 BMI group and risk of relapse, treatment‐related mortality, and overall mortality

Within the follow‐up period, 324 patients suffered a first relapse, 102 died from treatment‐related causes, and 244 died for any reason. Of the 102 patients that died from treatment‐related causes, 42 died without previous hematopoietic stem cell transplantation or relapse, 14 died after hematopoietic stem cell transplantation in first complete remission and 46 died after relapse from causes other than disease progression. In two cases, reason for death was unknown (excluded from treatment‐related mortality analyses). The remaining 140 deaths were due to disease progression.

**Figure 2:**
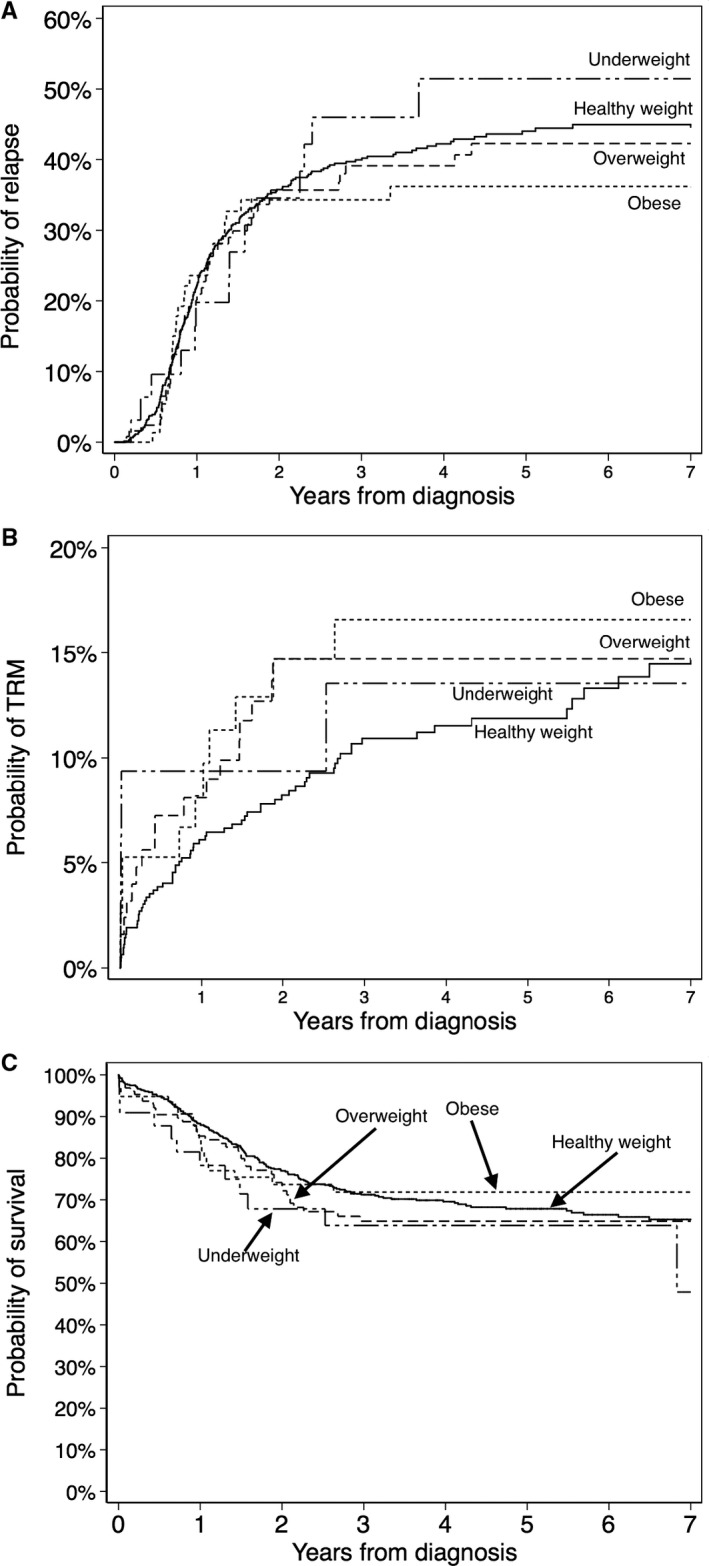
Cumulative incidence of relapse, treatment‐related mortality, and overall survival according to body mass index group. A, Cumulative incidence of relapse. B, Cumulative incidence of treatment‐related mortality (TRM). C, Kaplan‐Meier estimator of overall survival

Overall, in the cohort the 5‐year cumulative incidence of relapse was 43% (95%‐confidence interval (CI) 40‐47), the 5‐year cumulative incidence of treatment‐related mortality was 13% (95% CI 10‐15), and 5‐year overall survival was 67% (95% CI 64‐71). The results for associations between BMI group and risk of relapse, treatment‐related mortality, and overall mortality are presented in Table 2 and Figure 2.

**Table 2:** Relapse and survival according to body mass index groups

	Underweight (n = 32)	Healthy weight (n = 632)	Overweight (n = 127)	Obese (n = 76)
Relapse				
N	14	239	46	25
5‐y cumulative incidence (95% CI)	52% (32‐73%)	44% (40‐48%)	42% (32‐52%)	36% (24‐48%)
Crude HR (95% CI)	1.3 (0.7‐2.1)	1	1.0 (0.7‐1.3)	0.9 (0.6‐1.3)
Adjusted HR (95% CI)^a^	1.2 (0.7‐2.0)	1	0.9 (0.7‐1.3)	0.7 (0.5‐1.1)
Treatment‐ related mortality				
N	4	69	17	12
5‐y cumulative incidence (95% CI)	13% (0‐26%)	12% (9‐15%)	15% (8‐21%)	16% (7‐25%)
Crude HR (95% CI)	1.2 (0.4‐3.3)	1	1.2 (0.7‐2.1)	1.5 (0.8‐2.7)
Adjusted HR (95% CI)^a^	1.2 (0.4‐3.3)	1	1.0 (0.6‐1.7)	1.1 (0.6‐2.1)
Overall mortality				
N	12	173	39	20
5‐y overall survival (95% CI)	63% (42‐77%)	68% (63‐72%)	65% (55‐73%)	71% (59‐81%)
Crude HR (95% CI)	1.5 (0.8‐2.6)	1	1.1 (0.8‐1.6)	1.0 (0.6‐1.6)
Adjusted HR (95% CI)^a^	1.4 (0.8‐2.5)	1	1.0 (0.7‐1.4)	0.8 (0.5‐1.3)

Note

Underweight: <−2 SD, Healthy weight: −2 to +2 SD for age 2‐5 and −2 to +1 SD for age 6‐17, Overweight: >2‐3 SD for age 2‐5 and >1‐2 SD for age 6‐17, Obesity >3 SD for age 2‐5 and >2 SD for age 6‐17.

Abbreviations: CI: confidence interval, HR: hazard ratio

a Adjusted for age (continuously), sex, year of diagnosis (continuously), and country group (Nordic countries, Belgium/the Netherlands, Hong Kong, or Canada/USA).

We found no evidence of associations between BMI group and risk of relapse, risk of treatment‐related mortality, or risk of death for any reason (Figure 2, Table 2, and Supporting Information). Including cytogenetic abnormalities in the models, as mediation analyses did not change the results (Table S2). Combining the overweight and obese group, we saw similar results with no evidence of association between overweight/obesity and risk of relapse, risk of treatment‐related mortality, or risk of death for any reason (Table S3).

### 3.2 BMI group and cytogenetic abnormalities

**Figure 3:**
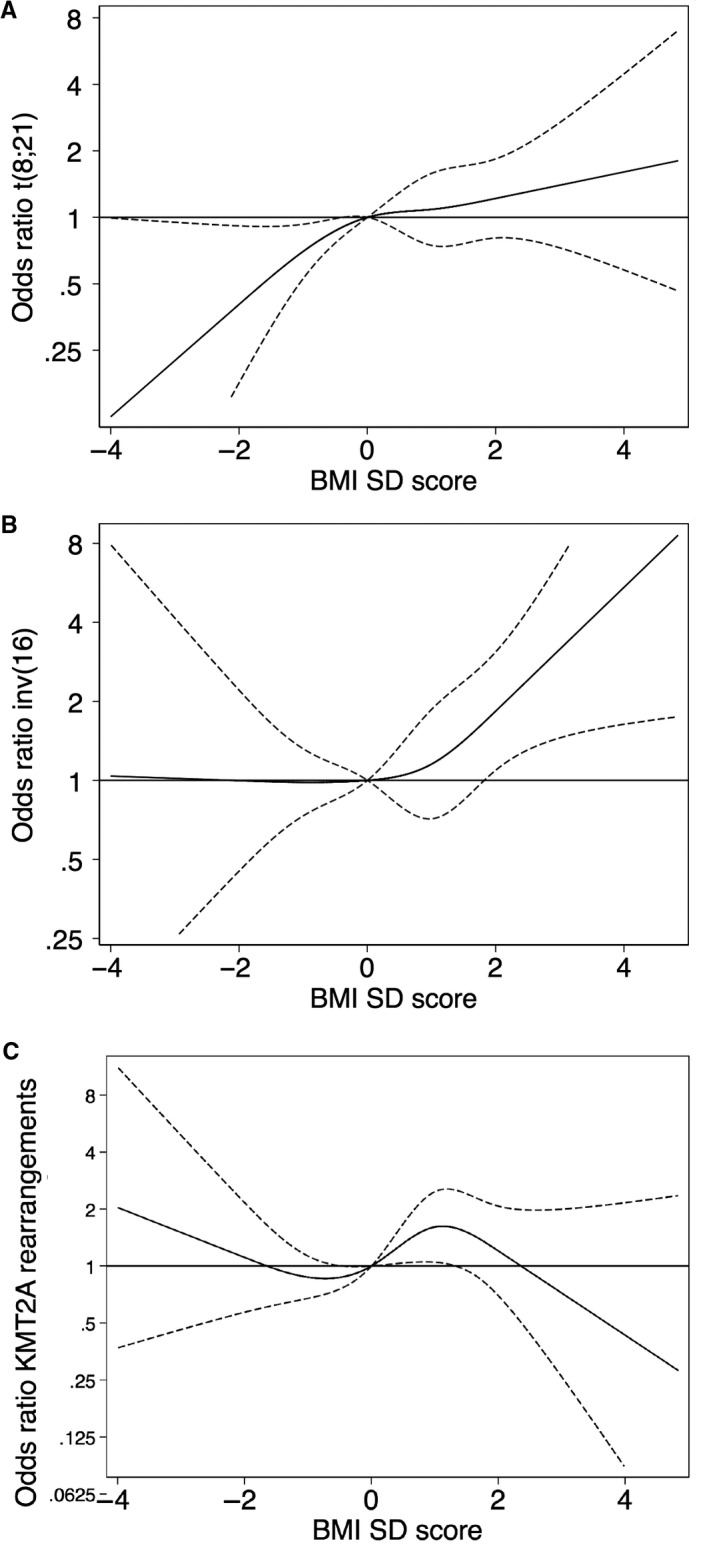
Associations of body mass index standard deviation score and cytogenetic abnormalities. Restricted cubic splines for the association of body mass index standard deviation (BMI SD) score and odds ratio for three different cytogenetic abnormalities: (A) t(8;21), (B) inv(16)/t(16;16), and (C) *KMT2A* rearrangements. The full line is the estimate and the dotted lines, the 95% confidence interval. Estimates are adjusted for age, sex and country group (Nordic countries, Belgium/the Netherlands, Hong Kong, or Canada/USA). The y‐axis is log‐scale. The reference for all plots is set at body mass index standard deviation score of 0

In 36 (4.2%) cases, information on t(8;21) and inv(16) were missing, and in 38 (4.4%) cases information on *KMT2A* rearrangements were missing. In the remaining cases, 155 (19%) of patients had t(8;21), 80 (10%) had inv(16) and 100 (12%) had a *KMT2A* rearrangement. Patients with *KMT2A* rearrangements were younger, the three cytogenetic abnormality groups showed a different pattern of white blood cell count at diagnoses and fewer with the cytogenetic abnormalities were transplanted in first complete remission compared to the entire cohort (Table S4). The results for associations between BMI group and cytogenetic abnormalities are presented in Table 3. The frequency and odds ratio of t(8;21) and inv(16) increased with increasing BMI group (Table 3). For *KMT2A* rearrangements, the association with BMI group was less clear: overweight cases had higher frequency and obese cases had a lower frequency (Table 3). Figure 3 shows the association between BMI standard deviation score examined continuously and the adjusted odds ratio for cytogenetic abnormalities.

**Table 3:** Cytogenetic abnormalities according to body mass index groups

	Underweight	Healthy weight	Overweight	Obese
t(8;21)				
N (%)	2 (7)	106 (17)	26 (21)	21 (31)
Crude OR (95% CI)	0.3 (0.08‐1.4)	1	1.2 (0.8‐2.0)	2.1 (1.2‐3.7)
Adjusted OR (95% CI)^a^	0.3 (0.08‐1.4)	1	1.2 (0.7‐1.9)	1.9 (1.1‐3.4)
inv(16)/t(16;16)				
N (%)	1 (3)	53 (9)	14 (11)	12 (18)
Crude OR (95% CI)	0.4 (0.05‐2.7)	1	1.3 (0.7‐2.5)	2.2 (1.1‐4.4)
Adjusted OR (95% CI)^a^	0.3 (0.05‐2.6)	1	1.6 (0.8‐3.0)	2.8 (1.3‐5.8)
*KMT2A* rearrangements				
N (%)	5 (17)	70 (12)	22 (18)	3 (4)
Crude OR (95% CI)	1.5 (0.6‐4.1)	1	1.7 (1.0‐2.8)	0.4 (0.1‐1.2)
Adjusted OR (95% CI)^a^	1.8 (0.6‐4.9)	1	2.0 (1.1‐3.5)	0.5 (0.1‐1.5)

Note

Underweight: <−2 SD, Healthy weight: −2 to +2 SD for age 2‐5 and −2 to +1 SD for age 6‐17, Overweight: >2‐3 SD for age 2‐5 and >1‐2 SD for age 6‐17, Obesity >3 SD for age 2‐5 and >2 SD for age 6‐17.

Abbreviations: CI: confidence interval, OR: odds ratio.

a Adjusted for age (continuously), sex and country group (Nordic countries, Belgium/the Netherlands, Hong Kong, or Canada/USA).

## 4 DISCUSSION

In this multinational study of 867 pediatric AML patients diagnosed within the last two decades, we found no evidence of associations between BMI group and risk of relapse, treatment‐related, or overall mortality. We found associations of obesity and a higher frequency of t(8;21) and inv(16).

We used the World Health Organization (WHO) Child Growth Standard for calculating BMI SD score based on age and sex, due to the multinational design of our study. The growth standard is based on data from children from six different countries (Brazil, Ghana, India, Norway, Oman and the USA) with different ethnic backgrounds and cultural settings^11^, ^19^ and therefore the standard is the most relevant when doing a multinational study. The WHO classification has been shown to have a higher prevalence of overweight and obesity but with similar trends when compared with the International Obesity Task Force and the US Centers for Disease Control and Prevention classifications.^20^

In contrast to two previous North American studies (including mainly patients from the US) that showed that overweight/obesity was associated with increased treatment‐related mortality,^4^, ^5^ we found no evidence of associations between BMI group and outcome (Figure 2 and Table 2). There could be several explanations for the difference in results. Supportive care improves over time, and while all patients in the two North American studies were treated up to 2008, approximately half the patients included in our study were treated after 2008. In a previous study, we showed that overweight children were at higher risk for toxicity,^6^ but the mortality rate from severe toxicity could have decreased in recent years. Both North American studies included patients up to 20 years of age, whereas this study only included patients up to 17 years of age (upper age limit for the pediatric AML protocols in the NOPHO‐DBH countries). The frequency of obesity and toxicity increases with age^4^, ^5^, ^6^ and therefore the difference in age distribution could be part of the explanation for difference in treatment‐related mortality. Due to our cohort consisting of patients treated on 17 different protocols, we did not find it feasible to stratify on protocol. Therefore it is possible that an association between BMI group and outcome exist for some treatment regimes. Finally it is important to note that our finding of no association between BMI group and outcome was based on a relatively small cohort and therefore could be a type II error (failure to reject a false null hypothesis). We may have failed to show an association between obesity and outcome because of the relatively low prevalence of obesity in this study, likely reflecting the prevalence of obesity in participating countries. If an association between BMI and outcomes does exist, its effect could be magnified in countries where the prevalence of obesity is higher. A future meta‐analysis may overcome the challenges related to small sample size and the relatively low prevalence of obesity in this study.

The frequency of cytogenetic abnormalities in our study was comparable to previous reported frequencies. The frequency of t(8;21) was slightly higher and the frequency of *KMT2A* rearrangement slightly lower compared to what was previously published,^21^, ^22^ but this was probably due to these abnormalities being age‐dependent and the fact that children below 2 years were excluded from this study. Increasing BMI standard deviation score was associated with increasing frequency of inv(16) and t(8;21) (Table 3 and Figure 3). This is the first rapport of associations of BMI standard deviation score and cytogenetic abnormalities in children, but in adults, obesity has been associated with AML with recurrent cytogenetic abnormalities (including t(8,21) and inv(16)/t(16;16)), though the study did not specify which cytogenetic abnormalities.^23^ Our results indicate that either a common etiology for overweight and certain types of AML exist or that obesity is associated with developing certain leukemic cytogenetic abnormalities.

A common variant in the gene coding for the fat mass‐ and obesity‐associated protein (FTO*)* is known to predispose to obesity in adults and children.^24^ Recently, it has been shown that FTO, an RNA demethylase, plays an oncogenic role in AML in adults.^25^ It might therefore be biologically plausible that obesity and (certain types of) AML could have a common etiology in children as well.

If, instead, obesity is associated with developing core‐binding factor leukemia, it is possible that obese children have a higher risk of developing AML, comparable to obese adults.^26^, ^27^ A recent study showed that bone marrow adipocytes support the survival and proliferation of AML blasts.^28^ Also, there is growing evidence that higher birth‐weight is associated with developing both acute lymphoid leukemia and AML in childhood and young adulthood,^29^ but studies of associations between birth‐weight and cytogenetic subgroups of AML are lacking. However, it is important to note, that this study was not designed to examine the effect of obesity on the risk of developing leukemia. Studies of incidence of AML in overweight children compared to healthy weight children are needed in general, and with a focus on specific cytogenetic subtypes in particular. As opposed to our study, future studies would also benefit from collecting BMI SD score at several time points before AML diagnosis.

Ethnicity could be a confounder in this study. Gramatges et al demonstrated a higher frequency of t(8;21) in Hispanic children with AML compared to non‐Hispanic whites.^30^ However, the study did not show frequency of overweight in the two groups, and Hispanic children are more overweight in the US.^31^ We did not have information on ethnicity for about half our cases, and because of our multinational cohort, ethnicity groups were difficult to define. Therefore, we chose not to include ethnicity in our models. As a very crude marker for ethnicity, we adjusted cytogenetic abnormality analyses for country group and did not find considerable differences with crude estimates (Table 3). Environmental factors could also be a cause of both overweight and cytogenetic abnormalities, but knowledge is lacking.

We did not find evidence that the increased frequency of core‐binding factor leukemia with increasing BMI standard deviations score led to improved outcome. Yet, although the estimates are quite imprecise with wide confidence intervals, we did observe a decreased risk of relapse with increasing BMI standard deviation score (Table 2 and Figure S1).

Our study needs to be interpreted in light of certain limitations. We only had information on BMI at diagnosis and therefore could not investigate effect of weight change on outcome, a factor which has been associated with outcome in pediatric acute lymphoblastic leukemia.^32^, ^33^, ^34^ The North American cohort was created for studies on infectious complications in pediatric AML,^10^, ^35^, ^36^, ^37^ and not for studies on outcome and therefore follow‐up is short for some patients. The patients included in the study were treated within a rather large timeframe (1995‐2016) on 17 different protocols, which makes the cohort relatively heterogeneous. However, despite differences in protocols they all included cytarabine (cumulative dose from 20 to 85 g/m^2^), one or more anthracyclines (daunorubicin/idarubicin or mitoxantrone or both), and most included etoposide. We did not have information on socioeconomic status. Socioeconomic status has been shown to be associated with risk of overweight in western developed countries^38^ and outcome from pediatric AML in the USA.^39^ But since healthcare costs are tax‐covered for almost all included countries (only 17 included patients were from USA), the effect of socioeconomic status on outcome may be smaller in our cohort, than in the USA. We speculate that the effect of socioeconomic status on outcome in the USA could be part of the explanation for the discrepancy in results between this and the two previous North American studies.^4^, ^5^

In conclusion, in this large multinational cohort, we found that, in children with AML, overweight and obesity were not associated with outcome, which is in contrast to previous studies. We found that obesity was associated with a higher frequency of core‐binding factor leukemia. This finding raises the question whether overweight in children increases the risk of developing core‐binding factor AML, but further studies are needed.

## CONFLICT OF INTEREST

The authors have no conflicts of interest.

## AUTHOR CONTRIBUTIONS

DJAL, LS, and HH designed the project; DJAL, PHA, LS, and HH interpreted the data, and drafted the manuscript; DJAL performed statistical analyses; JA, GJLK, SY.H., OGJ, MK, BL, BDM, JP, and BZ collected the data and contributed to the writing of the manuscript.

## Data Availability

The data that support the findings of this study are available on request from the corresponding author. The data are not publicly available due to privacy or ethical restrictions.
