# Supplementary material for: Associations between pretherapeutic body mass index, outcome, and cytogenetic abnormalities in pediatric acute myeloid leukemia
Source: Cancer Med. 2019 Sep 18;8(15):6634–43. doi: 10.1002/cam4.2554 (PMC6825997; doi:10.1002/cam4.2554)
Supplement: Supplementary file 1 [file CAM4-8-6634-s001.doc]

**Supporting information figure S1:**

**Associations of body mass index standard deviation score and risk of relapse, treatment-related mortality, and overall mortality**

Graphs show the continuous associations of BMI standard deviation score and hazard ratio for outcomes: (A) relapse (B) treatment-related mortality (TRM) (C) overall mortality. The full line shows the estimate and the dotted lines, the 95% confidence interval. All analyses were adjusted for age, sex, country group and year of diagnosis. The y-axis is log-scale. The reference for all plots is set at 0. Plots were created using restricted cubic splines with four knots at BMI SD score -2, 0, 1, and 2.

**Supporting information table S2:**

**Association of body mass index group and outcome with cytogenetic abnormalities included in model**

We did not include cytogenetic abnormalities in the models of BMI group and relapse, treatment-related mortality and overall mortality, because cytogenetic abnormalities do not influence BMI group. Therefore, according to the definition, cytogenetic abnormalities are not confounders.

But cytogenetic abnormalities could be a mediator in the association of BMI group and relapse, treatment-related mortality and overall mortality and therefore we did a mediation analysis including the variables. The analysis did not change the results markedly:

|  |  | Underweight | Healthy weight | Overweight | Obese |
| --- | --- | --- | --- | --- | --- |
| Relapse | Crude HR (95%-CI) | 1.3 (0.7–2.1) | 1 | 1.0 (0.7–1.3) | 0.9 (0.6–1.3) |
|  | Adjusted HR (95%-CI)^†^ | 1.2 (0.7–2.0) | 1 | 0.9 (0.7–1.3) | 0.7 (0.5–1.1) |
|  | MA: Adjusted HR (95%-CI)^$^ | 1.0 (0.6–1.8) | 1 | 0.9 (0.7–1.3) | 0.8 (0.5–1.3) |
| Treatment- related mortality | Crude HR (95%-CI) | 1.2 (0.4–3.3) | 1 | 1.2 (0.7–2.1) | 1.5 (0.8–2.7) |
|  | Adjusted HR (95%-CI)^†^ | 1.2 (0.4–3.3) | 1 | 1.0 (0.6–1.7) | 1.1 (0.6–2.1) |
|  | MA: Adjusted HR (95%-CI)^$^ | 1.2 (0.4–3.5) | 1 | 1.0 (0.5–1.7) | 1.2 (0.6–2.4) |
| Overall mortality | Crude HR (95%-CI) | 1.5 (0.8–2.6) | 1 | 1.1 (0.8–1.6) | 1.0 (0.6–1.6) |
|  | Adjusted HR (95%-CI)^†^ | 1.4 (0.8–2.5) | 1 | 1.0 (0.7–1.4) | 0.8 (0.5–1.3) |
|  | MA: Adjusted HR (95%-CI)^$^ | 1.1 (0.6–2.1) | 1 | 1.0 (0.7–1.4) | 0.9 (0.5–1.5) |

Underweight: <-2 SD, Healthy weight: -2–+2 SD for age 2–5 and -2–+1 SD for age 6–17,

Overweight: >2–3 SD for age 2–5 and >1–2 SD for age 6-17, Obesity >3 SD for age 2–5 and >2 SD for age 6–17

CI: confidence interval, HR: hazard ratio, MA: Mediation analysis

† Adjusted for age (continuously), sex, year of diagnosis (continuously), and country group (Nordic countries, Belgium/the Netherlands, Hong Kong or Canada/USA).

$ Adjusted for age (continuously), sex, year of diagnosis (continuously), and country group (Nordic countries, Belgium/the Netherlands, Hong Kong or Canada/USA), **core-binding factor leukemia and *KMT2A* rearrangements**.

**Supporting information table S3: Relapse and survival according to BMI groups with overweight and obesity combined**

Underweight: <-2 SD, Healthy weight: -2–+2 SD for age 2–5 and -2–+1 SD for age 6–17,

Overweight/obesity: >2 SD for age 2–5 and >1 SD for age 6-17.

CI: confidence interval, HR: hazard ratio

|  |  | Healthy weight (n=632) | Overweight/obesity  (n=203) |
| --- | --- | --- | --- |
| Relapse | Crude HR (95%-CI) | 1 | 0.9 (0.7–1.2) |
|  | Adjusted HR (95%-CI)^†^ | 1 | 0.8 (0.6–1.1) |
| Treatment-related mortality | Crude HR (95%-CI) | 1 | 1.3 (0.9–2.0) |
|  | Adjusted HR (95%-CI)^†^ | 1 | 1.1 (0.7–1.7) |
| Overall mortality | Crude HR (95%-CI) | 1 | 1.1 (0.8–1.5) |
|  | Adjusted HR (95%-CI)^†^ | 1 | 0.9 (0.7–1.2) |

† Adjusted for age (continuously), sex, year of diagnosis (continuously), and country group (Nordic countries, Belgium/the Netherlands, Hong Kong or Canada/USA).

**Supporting information table S4: Baseline characteristics according to cytogenetic abnormalities**

WBC: white blood count

|  |  | Total cohort  N (%) | t(8;21)^†^  N (%) | Inv(16)/t(16;16)^†^  N (%) | *KMT2A* rearrangements^†^  N (%) |
| --- | --- | --- | --- | --- | --- |
| Patients |  | 867 (100) | 155 (19) | 80 (10) | 100 (12) |
| Sex | Male | 462 (53) | 89 (57) | 47 (59) | 52 (52) |
|  | Female | 405 (47) | 66 (43) | 33 (41) | 48 (48) |
| Age | Median (range) | 10 (2–17) | 11 (2–17) | 10 (2–17) | 7 (2–17) |
| Country group | The Nordic countries | 266 (31) | 55 (35) | 28 (35) | 37 (37) |
|  | The Netherlands and Belgium | 140 (16) | 24 (15) | 16 (20) | 7 (7) |
|  | Hong Kong | 50 (6) | 5 (3) | 6 (8) | 6 (6) |
|  | Canada and USA | 411 (47) | 71 (46) | 30 (38) | 50 (50) |
| WBC at diagnosis^†^ | 0-9.9 | 300 (35) | 51 (33) | 14 (18) | 44 (44) |
|  | 10-99.9 | 428 (49) | 101 (65) | 51 (64) | 33 (33) |
|  | ≥100 | 137 (16) | 2 (1) | 15 (19) | 23 (23) |
| Year of diagnosis | 1995-1999 | 117 (13) | 16 (10) | 5 (6) | 6 (6) |
|  | 2000-2004 | 130 (15) | 25 (16) | 7 (9) | 19 (9) |
|  | 2005-2009 | 240 (28) | 51 (33) | 25 (31) | 29 (29) |
|  | 2010-2014 | 303 (35) | 52 (34) | 35 (44) | 36 (36) |
|  | 2015-2016 | 77 (9) | 11 (7) | 8 (10) | 10 (10) |
| Stem cell transplanted in first complete remission | | 224 (26) | 17 (11) | 6 (8) | 17 (17) |

† Data on WBC missing in 2 cases, data on t(8;21) and inv(16) missing in 36 cases, and data on *KMT2A* rearrangements missing in 38 cases.
